# A rare sugar, allose, inhibits the development of *Plasmodium* parasites in the *Anopheles* mosquito independently of midgut microbiota

**DOI:** 10.3389/fcimb.2023.1162918

**Published:** 2023-07-20

**Authors:** Daiki Mizushima, Daisuke S. Yamamoto, Ahmed Tabbabi, Meiji Arai, Hirotomo Kato

**Affiliations:** ^1^ Division of Medical Zoology, Department of Infection and Immunity, Jichi Medical University, Tochigi, Japan; ^2^ Department of International Medical Zoology, Faculty of Medicine, Kagawa University, Kita-gun, Kagawa, Japan

**Keywords:** allose, microbiota, midgut, *Anopheles*, *Plasmodium*, luciferase assay, next-generation sequencing

## Abstract

A rare sugar, allose, was reported to inhibit the development of *Plasmodium* parasites in *Anopheles* mosquitoes; however, the mechanism remains unknown. The present study addressed the inhibitory mechanism of allose on the development of the *Plasmodium* parasite by connecting it with bacteria involvement in the midgut. In addition, further inhibitory sugars against *Plasmodium* infection in mosquitoes were explored. Antibiotic-treated and antibiotic-untreated *Anopheles stephensi* were fed fructose with or without allose. The mosquitoes were infected with luciferase-expressing *Plasmodium berghei*, and parasite development was evaluated by luciferase activity. Bacterial composition analysis in gut of their mosquitoes was performed with comprehensive *16S ribosomal RNA* sequencing. As the result, allose inhibited the development of oocysts in mosquitoes regardless of prior antibiotic treatment. Microbiome analysis showed that the midgut bacterial composition in mosquitoes before and after blood feeding was not affected by allose. Although allose inhibited transient growth of the midgut microbiota of mosquitoes after blood feeding, neither toxic nor inhibitory effects of allose on the dominant midgut bacteria were observed. Ookinete development in the mosquito midgut was also not affected by allose feeding. Additional 15 sugars including six monosaccharides, four polyols, and five polysaccharides were tested; however, no inhibitory effect against *Plasmodium* development in mosquitoes was observed. These results indicated that allose inhibits parasite development in midgut stage of the mosquito independently of midgut microbiota. Although further studies are needed, our results suggest that allose may be a useful material for the vector control of malaria as a “transmission-blocking sugar.”

## 1 Introduction

Malaria is a life-threatening disease caused by *Plasmodium* parasites, with the estimated number of deaths reaching 627,000 per year in 2020 (World Health Organization (WHO), 2021). *Plasmodium* parasites are transmitted by *Anopheles* mosquitoes, ubiquitously distributing in endemic areas (Moffett et al., 2007; Autino et al., 2012). Vector control is a cornerstone in malaria control, owing to the lack of reliable vaccines, the emergence of drug resistance, and unaffordable potent antimalarials. Studies on transmission-blocking strategies, which aim to interrupt the life cycle of the *Plasmodium* species within mosquitoes, are progressing, and elucidation of more detailed transmission mechanisms including interaction between *Anopheles* mosquitoes and *Plasmodium* is expected.

The midgut of the mosquito is important not only for digestion and uptake of nutrients but also as the entry site of pathogens including *Plasmodium* parasites. When *Anopheles* mosquitoes ingest *Plasmodium*-infected blood from the host, parasites mate and develop into motile ookinetes within 16–20 h (Blandin et al., 2004). Ookinetes traverse across the midgut epithelium within 24 h after the feeding and transform into oocysts on the basal side of the midgut (Baton and Ranford-Cartwright, 2004; Blandin et al., 2004). Sporozoites develop and proliferate within oocysts over about the next 10 days. The sporozoites released from oocysts invade the salivary glands, in which they wait for transmission to the next host via a mosquito bite (Blandin et al., 2004; Frischknecht et al., 2004). The early phase of the parasite infection, from ingestion to invasion into midgut epithelium, is the bottleneck for the parasite in the mosquito cycle and, therefore, is considered to be a suitable target for a transmission-blocking strategy.

Mosquitoes develop protective mechanisms against infectious agents in the midgut, which include the immune system and biological barriers. Parasite invasion and development in the midgut are affected by immune-related factors such as thioester-containing protein 1 (TEP1) (Blandin et al., 2004; Osta, 2004; Dong et al., 2006; Baxter et al., 2010; Povelones et al., 2011) and antimicrobial peptides such as gambicin regulated by the immune deficiency (IMD) pathway through transcription factor Rel2 (Vizioli et al., 2001; Dong et al., 2006), as well as nitric oxide and reactive oxygen species (ROS) induced by invasion of gut epithelium (Luckhart et al., 1998; Molina-Cruz et al., 2008). In addition, malaria parasites are continuously exposed to microbes in the mosquito gut. This naturally acquired microbial flora can modulate the mosquito’s vectorial capacity. Comprehensive functional approaches have been undertaken to elucidate the interplay between commensal bacteria and the development of the malaria parasite in its natural vector. Mosquitoes treated with antibiotics, which diminish bacteria quantity, are known to be more susceptible to *Plasmodium* parasite infection (Dong et al., 2009). Administration of some characterized bacteria, such as *Enterobacter* sp. and *Serratia marcescens*, reduced the development of *Plasmodium* oocysts by eliciting anti-*Plasmodium* immunity in mosquitoes (Cirimotich et al., 2011; Bando et al., 2013; Bahia et al., 2014).

In nature, both male and female *Anopheles* mosquitoes ordinarily ingest sugars from floral nectar, but only females suck blood at a time of reproduction. Sugars function not only as an energy source but also as prebiotics for gut microbiota. As examples, oligosaccharides, which are composed of two to 10 monosaccharides, improve the host gastrointestinal condition by promoting beneficial microbiota (Mano et al., 2018), and polyols and hydrogenated carbohydrates are known to affect the ecology of oral microflora and exhibit a cariostatic effect in mammals (Msomi et al., 2021). Sucrose, an oligosaccharide, maintains antiviral immunity in the gut of *Aedes aegypti* (Almire et al., 2021). A recent study reported that glucose induces the expansion of *Asaia bogorensis*, a commensal bacterium in the mosquito gut, that remodels glucose metabolism, resulting in the promotion of *Plasmodium* gametogenesis and parasite infection by increasing the pH of the mosquito midgut (Wang et al., 2021). However, a rare sugar, allose, which has been shown to influence ROS generation in mammalian and plant cells (Murata et al., 2003; Kimura et al., 2005; Kano et al., 2013), was shown to inhibit *Plasmodium* infection in mosquitoes (Tokuda et al., 2015). Currently, it remains unknown how allose inhibits malarial parasite development and whether it affects commensal bacterium in the mosquito gut.

The present study addressed the inhibitory mechanism of allose on the development of the *Plasmodium* parasite and the involvement of the microbiota in midgut fed allose in its mechanism. In addition, other inhibitory sugars potentially affecting the development of *Plasmodium* were explored.

## 2 Materials and methods

### 2.1 Mosquito and malaria parasite maintenance

*Anopheles stephensi* SDA-500 were maintained at 26°C with 60%–80% relative humidity under the condition of 13-h light/11-h dark at the Jichi Medical University. Larvae were fed with fish food, Hikari (KYORIN Co., Ltd., Hyogo, Japan). Adults were fed on filter paper soaked with 8% fructose (FUJIFILM Wako, Osaka, Japan). Luciferase-expressing *P. berghei* ANKA234 under the control of elongation factor 1-α promoter (*Pb-*luc) was injected into an Institute of Cancer Research (ICR) mouse intraperitoneally (Matsuoka et al., 2015). The mouse was intraperitoneally administered 100 µL of pyrimethamine (1 mg/mL) daily. Infected blood was stained with Giemsa’s stain solution (Merck KGaA, Darmstadt, Germany) to determine parasitemia. All procedures were carried out in accordance with the ethical guidelines, and this study was approved by the Institutional Animal Experiment Committee of the Jichi Medical University (approval numbers: 20003-01).

### 2.2 Sugar feeding, antibiotic treatment, and infection of *An stephensi* with *P berghei*

Sixteen sugars—seven monosaccharides (D-glucose, D-fructose, D-galactose, L-sorbose, D-mannose, D-xylose, and D-allose), four polyols (D-sorbitol, xylitol, erythritol, and maltitol), and five oligosaccharides (sucrose, D-lactose, lactulose, D-maltose, and D-raffinose) (Tokyo Chemical Industry Co., Ltd., Tokyo, Japan)—were used in this study to evaluate their inhibitory effects on the development of *Plasmodium*. Of these, D-allose, erythritol, L-sorbose, and xylitol are categorized as rare sugars as they are rarely present in nature. Mosquitoes were fed 8% fructose with 100 mM of each abovementioned sugar for 24 h *ad libitum* and fasted for the following 24 h. The mosquitoes were allowed to feed on *Pb*-luc–infected mice showing 1.5%–2.0% parasitemia for 30 min, and unfed and partially blood-fed mosquitoes were removed from cages. Fully engorged mosquitoes were maintained on 8% (444 mM) fructose containing 100 mM of each sugar for an additional 10 days at 21°C. The concentration of additional sugar was matched with the effective concentration of allose (100 mM) (Tokuda et al., 2015). For the antibiotic treatment, mosquitoes were fed 8% fructose with 100 mM allose containing penicillin (200 U/mL) and streptomycin (200 µg/mL; Thermo Fisher Scientific Inc., Waltham, MA, USA) for 24 h *ad libitum*. After starvation for the following 24 h, the mosquitoes were allowed to feed on *Pb*-luc–infected mice showing 10% parasitemia for 30 min. The mosquitoes were maintained as mentioned above. Infection of Pb-luc in mosquitoes fed with the infected blood after 10 days was determined by measuring luciferase activity and by counting oocysts in the midgut (Matsuoka et al., 2015).

### 2.3 Luciferase assay

Luciferase activity was measured using a Luciferase assay kit according to the manufacturer’s protocol (Promega Inc., Madison, WI, USA). Briefly, the whole body of mosquitoes was individually homogenized with disposable homogenizer in a 1.5-mL tube containing cell lysis buffer. The homogenized samples were centrifuged at 15,000 × *g* for 1 min, and 10 µL of each supernatant was mixed with 50 µL of substrate in a 96-well black microplate. Luminescence was measured by a SpectraMax^®^ M5 microplate reader (Molecular Devices, LLC., San Jose, CA, USA).

### 2.4 Effect of allose on *Plasmodium* parasite *ex vivo*

To evaluate the effect of allose on ookinete development, mosquitoes were dissected at 24 h after infection, and midguts were collected. The midgut containing ookinetes was suspended individually in Roswell Park Memorial Institute (RPMI) 1640 medium (Nakarai Tesque Inc., Kyoto, Japan) supplemented with 20% heat-inactivated fetal calf serum, penicillin (100 U/mL), streptomycin (100 µg/mL; Thermo Fisher Scientific Inc.), and 25 mM Hydroxyethylpiperazine ethane sulfonic acid (HEPES) (pH 7.4; Tokyo Chemical Industry Co., Ltd.). The suspension was fixed and stained with Giemsa’s stain solution, and, then, ookinetes were counted under a microscope. The number of red blood cells (RBCs) in each sample was also counted to compensate the amount of ingested blood in each mosquito, and the ookinete count per 10^6^ RBCs was determined.

### 2.5 Midgut sample collection and DNA extraction for microbiota analysis

Mosquitoes were collected on days 1, 3, and 7 post-infection with *Pb*-luc at 21°C and prior to the infection as a control. They were anesthetized on ice, sterilized with 70% ethanol containing 1% hypochlorite, and then rinsed with phosphate-buffered saline (PBS). Midguts were taken out and washed with sterilized PBS (−), and 10 midguts were pooled in a 2-mL tube containing 0.5 mL of PBS with 0.1-mm Zirconia/Silica beads (Bio Spec Products Inc., Bartlesville, OK, USA). The midgut samples containing microbiota in the gut were mechanically disrupted by a bead beater (TAITEC Co., Saitama, Japan) at 3,500 rpm for 1 min, and DNA was purified using the ReliaPre DNA Clean-Up and Concentration System (Promega Inc.).

### 2.6 *16S ribosomal RNA*–based metagenomic analysis of mosquito midgut microbiota

Bacterial *16S ribosomal RNA* (*16S rRNA*) fragments containing hypervariable V3–V4 region were amplified, and index sequences were added by PCR with Tks Gflex DNA polymerase (Takara Bio Inc., Shiga, Japan) using primer sets according to the manufacturer’s protocol (https://jp.support.illumina.com/downloads/16s_metagenomic_sequencing_library_preparation.html ; accessed on 1 February, 2022). The amplified fragments were purified using a FastGene PCR/Gel extraction kit (Nippon Genetics Co., Ltd., Tokyo, Japan), and the concentration was quantified using a Qbit3 Fluorometer with Qbit dsDNA HS assay kit (Thermo Fisher Scientific Inc.). Paired-end sequencing analysis was conducted on the MiSeq^®^ platform with MiSeq Reagent Kit v3 for 600 cycles (Illumina, Inc., San Diego, CA, USA). Sequence data were trimmed and merged by DADA2 plugin in QIIME2 program version 2020.6 (Callahan et al., 2016; Bolyen et al., 2019). The sequences were clustered in amplicon sequence variants (ASVs) by feature-classifier plugin with the dataset of SILVA version 138 with ≥ 99% sequence identity (Quast et al., 2013; Yilmaz et al., 2014; Bokulich et al., 2018). To compare the diversities between groups, beta-diversity analyses with the Bray–Curtis dissimilarity index were performed with the q2-diversity plugin in QIIME2 at a sampling depth of 1,500 (Anderson, 2001).

### 2.7 Quantification of midgut bacteria in mosquitoes

Midgut bacteria in mosquitoes were quantified by quantitative PCR (qPCR) with KOD -SYBR- (TOYOBO Co., Ltd., Osaka, Japan) using Thermal Cycler Dice Real Time System Lite (Takara Bio Inc.). The bacterial *16S rRNA* was used as the target, and the *An. stephensi glyceraldehyde 3-phosphate dehydrogenase* (*Asgapdh*) was used as the reference. The primer sequences were 5′-ACHCCTACGGGDGGCWGCAG-3′ (16S-q-337F) and 5′-GTDTYACCGCGGYTGCTGGCAC-3′ (16S-q-514R) for amplification of the bacterial 16S rRNA gene and 5′-GCCGTCGGCAAGGTCATCCC-3′ (Asgapdh-q-F) and 5′-TTCATCGGTCCGTTGGCGGC-3′ (Asgapdh-q-R) for that of the *Asgapdh* (Yamamoto et al., 2016). Relative quantities of *16S rRNA* were determined by the comparative cycle threshold (ΔΔCt) method using the *Asgapdh* as the reference.

### 2.8 Isolation and identification of mosquito midgut bacteria

Mosquitoes were anesthetized on ice and washed with 70% ethanol containing 1% hypochlorite followed by sterilized PBS (−). The midgut was removed, rinsed with sterilized PBS (−), and homogenized in 20 µL of sterilized PBS (−). Ten microliters of the homogenate was spread on brain-heart infusion agar plate (Sigma Aldrich Inc., St. Louis, MO, USA), and the plate was incubated at 28°C for 3 days under aerobic and anaerobic conditions. For the identification of bacteria, the bacterial *16S rRNA* was amplified with KOD -One- (TOYOBO Co., Ltd.) using a pair of primers: 27F (5′-AGAGTTTGATCCTGGCTCAG-3′) and 1492R (5′-GGTTACCTTGTTACGACTT-3′) (Delong, 1992). The amplicon was purified using a FastGene PCR/Gel extraction Kit (Nippon Genetics Co., Ltd.), and the sequence was determined by the dideoxy chain termination method with a specific primer, 519F (5′-CAGCMGCCGCGGTAAT-3′), using a BigDye Terminator v.3.1 Cycle Sequencing Kit (Thermo Fisher Scientific Inc.). Homology was performed with the nucleotide BLAST tool (https://blast.ncbi.nlm.nih.gov/Blast.cgi), and a search was conducted of the taxonomic database (https://www.ncbi.nlm.nih.gov/taxonomy ) of the National Center for Biotechnology Information (NCBI) database.

### 2.9 Effect of allose on bacterial growth *in vitro*

A single colony of bacteria isolated from mosquito midgut was cultured in Luria-Bertani (LB) liquid medium at 28°C overnight. A part of the bacterial culture was transferred into LB liquid medium containing 8% fructose with 100 mM glucose or allose, and the optical density at a wavelength of 600 nm (OD_600_) of the suspensions was adjusted to 0.02 by spectrophotometer. The bacteria were further cultured at 28°C, and the growth was monitored for 36 h.

### 2.10 Sequence data availability

The microbiome sequencing data obtained in this study were registered in the NCBI/GenBank/ the DNA data bank of Japan (DDBJ) database under the accession numbers DRR337101-DRR337141. The *16S rRNA* sequences of *Leucobacter* sp. and *Phyllobacterium* sp. isolated from mosquito midgut are available under the accession numbers LC669551 and LC669552, respectively.

### 2.11 Statistical analysis

Statistical analyses were performed using R version 4.0.3. Welch’s *t*-test with Bonferroni’s modification was applied to ookinete counting, quantification of bacteria by qPCR, and evaluation of bacterial growth. Wilcoxon rank sum test with Bonferroni’s modification was conducted for the development of *Plasmodium* parasites within mosquitoes given different sugars. One-way analysis of variance with Holm’s modification was applied to assess the quantification of midgut bacterial amount the time-dependent changes of the relative abundance of the six dominant midgut bacteria genera.

## 3 Results

### 3.1 Allose inhibits development of *Plasmodium* parasite in the antibiotic-treated mosquito

**Figure 1:**
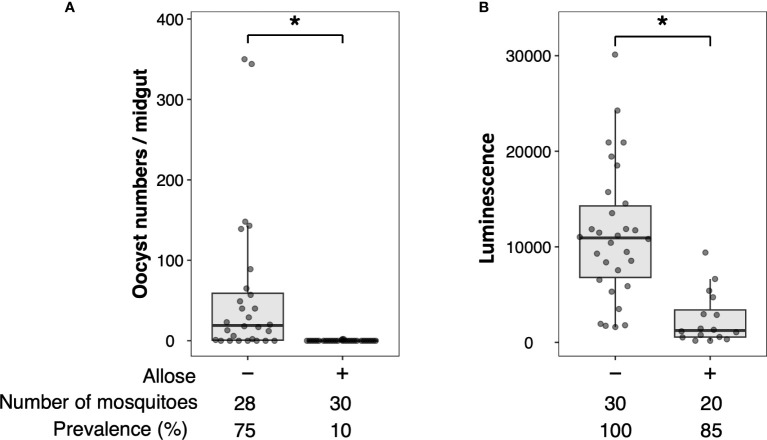
Allose inhibits oocyst development in the mosquito midgut. Allose-fed and allose-unfed mosquitoes were infected by luciferase-expressing *Plasmodium berghei*, and infectivity was determined 10 days post-infection by counting of the number of oocysts **(A)** and by measuring the luciferase activity **(B)**. The number of mosquitoes is indicated under each group. The prevalence of infection was calculated number of detected oocyst or luminescence sample divided by number of total sample. The results represent the standard deviation of the mean (SD) of biological replicates. Statistical significance was determined by Wilcoxon’s rank sum test with Bonferroni’s modification. *P < 0.01.

**Figure 2:**
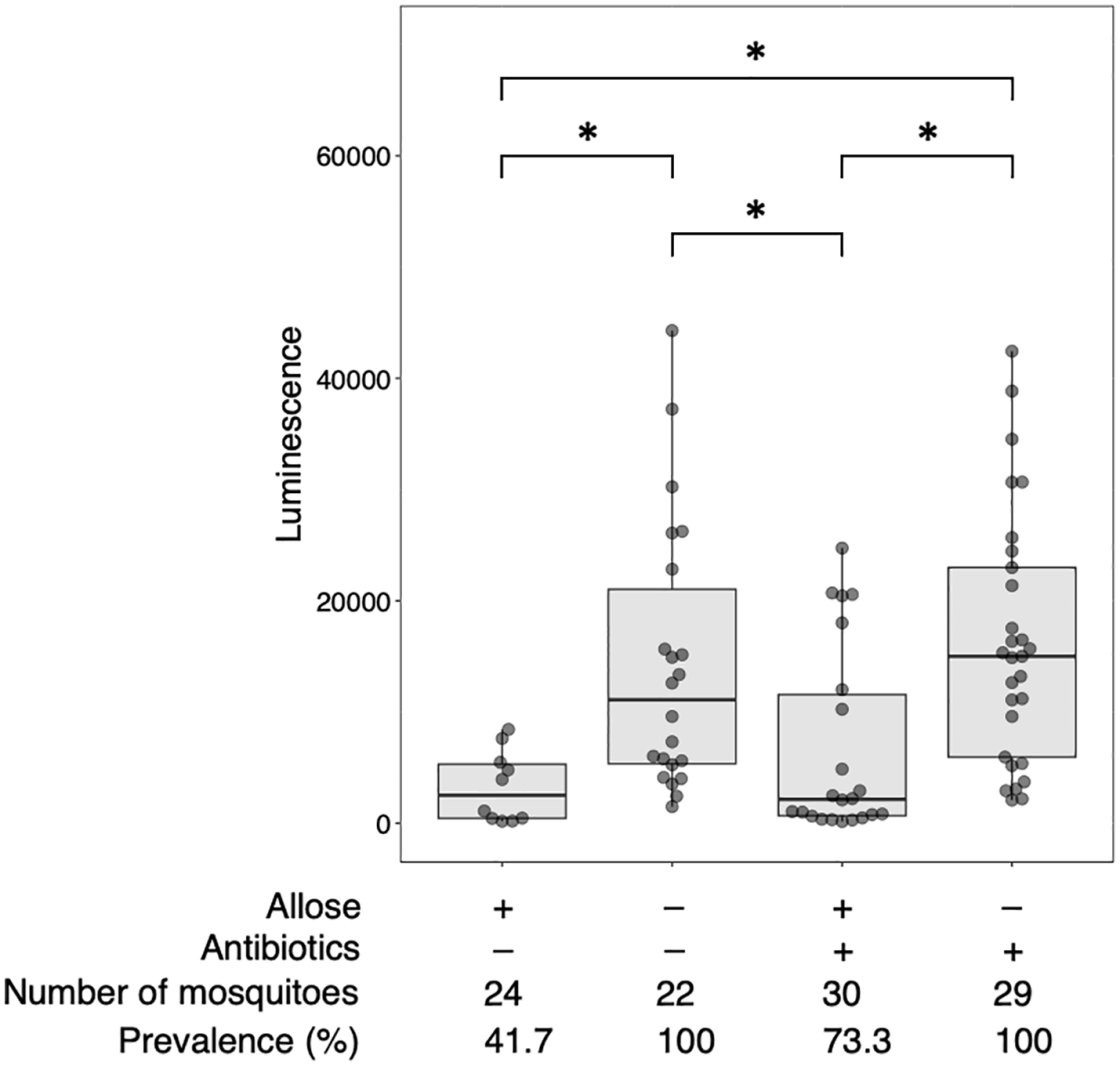
Allose inhibits the development of *Plasmodium* parasite in the mosquito by a midgut microbiota-independent mechanism. Antibiotic-treated and antibiotic-untreated mosquitoes were fed fructose with or without allose and then infected by luciferase-expressing *Plasmodium berghei*. Infectivity was determined by a luciferase assay. The number of mosquitoes is indicated under each group. The prevalence of infection was calculated number of detected oocyst or luminescence sample divided by number of total sample. The results represent the standard deviation of the mean (SD) of biological replicates. Statistical significance was determined by Wilcoxon’s rank sum test with Bonferroni’s modification. *P < 0.01.

Allose inhibits the development of *P. berghei* within *An. stephensi* by assessing the development of oocysts (Tokuda et al., 2015). To reconfirm this finding, luciferase activity was investigated in allose-fed mosquitoes infected with *Pb*-luc. As shown in **Figure 1**; **Supplementary Figure 4**, luciferase activities in the infected mosquitoes were consistent with oocyst counts at 10 days after *Pb*-luc infection. The inhibitory effect of allose was evaluated in mosquitoes treated with antibiotics, which have diminished microbiota in the midgut. Similar to untreated mosquitoes, the development of *Plasmodium* was inhibited by allose feeding in the antibiotic-treated mosquitoes (**Figure 2**). This result suggested that allose inhibits development of the *Plasmodium* parasite in the mosquito through a midgut microbiota-independent mechanism.

### 3.2 Allose affects the proliferation of midgut microbiota but not bacterial composition

**Figure 3:**
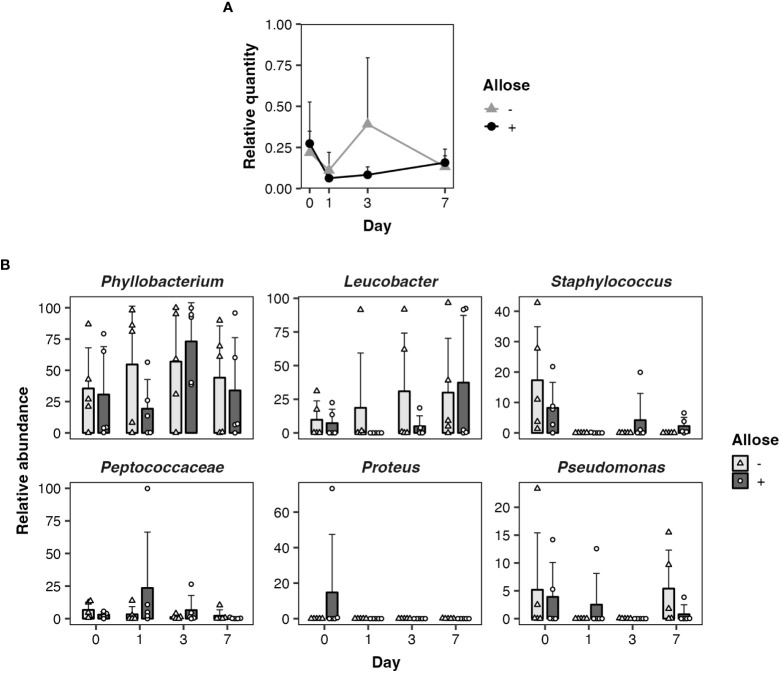
Allose affects the midgut quantity of bacteria but not their composition. Quantitative and qualitative changes of bacterial communities in the midgut were determined on days 0, 1, 3, and 7 after allose feeding. **(A)** Midgut bacteria of allose-fed (black circle) and allose-unfed (gray triangle) mosquitoes were quantified by qPCR. Relative quantities of the bacterial *16S rRNA* were normalized to the *An. stephensi glyceraldehyde 3-phosphate dehydrogenase* as the reference. Error bars indicate standard deviation (n = 5). **(B)** Midgut bacterial composition of allose-fed (open circle) and allose-unfed (open triangle) mosquitoes was determined by *16S rRNA*–based metagenomic analysis. The relative abundance of the six dominant genera—*Phyllobacterium*, *Leucobacter*, *Staphylococcus*, *Peptococcaceae*, *Proteus*, and *Pseudomonas*—are shown. The results are expressed as the mean titer and standard deviation for five biological experiments in each group.

As ingested sugars can affect midgut microbiota that may impact the development of parasites directly or indirectly, quantitative and qualitative changes of bacterial communities in the midgut of allose-fed mosquitoes were assessed. Midgut microbiota was quantified by qPCR. The relative quantity tended to be suppressed in allose-fed mosquitoes at 3 days after infection with no significant difference compared to allose-unfed mosquitoes and was comparable between groups on day 7 (**Figure 3A**). The decrease of bacterial quantities in the allose-fed group seemed to be attributed to inhibition of the rapid proliferation of gut bacteria initiated by blood feeding (**Figure 3A**). The composition of midgut bacteria was identified by *16S rRNA*–based metagenomic analysis and compared between allose-fed and allose-unfed mosquitoes. In total, 2,706,788 reads were obtained from 40 pooled samples, and *de novo* assembly of the reads generated 427 ASV. Of these, 113 ASVs consisting of 1,146,318 reads (42.4% of total reads) were not categorized in bacteria at the taxonomic level in the SILVA classifier, and, finally, 314 ASVs consisting of 1,497,066 reads (57.6%) were classified into families and genera of bacteria (**Supplementary Table S1**). The most abundant bacteria belong to the genus *Phyllobacterium* in the family *Rhizobiaceae* (51.4% of bacterial reads, 769,144 reads, 25 ASVs), followed by the genus *Leucobacter* in the family *Microbacteriaceae* (19.9% of bacterial reads, 297,535 reads, 12 ASVs), the genus *Staphylococcus* in the family *Staphylococcaceae* (3.5% of bacterial reads, 51,813 reads, 34 ASVs), the family *Peptococcaceae* (2.7% of bacterial reads, 40,358 reads, 23 ASVs), the genus *Proteus* in the family *Morganellaceae* (2.4% of bacterial reads, 35,621 reads, 7 ASVs), and the genus *Pseudomonas* in the family *Pseudomonadaceae* (2.3% of bacterial reads, 33,916 reads, 8 ASVs) (**Supplementary Table S1**). Other bacteria families consisting of less than 2% of reads were categorized as “others” in this study (17.9% of bacterial reads, 268,679 reads, 205 ASVs). More detailed information obtained by the metagenomic analysis is provided in **Supplementary Table S1**; **Figure 3B** shows the time-dependent changes of the relative abundance of the six dominant midgut bacteria genera. The most abundant genus, *Phyllobacterium*, consisted of 35.6%–57.0%, on average, in the allose-unfed group, and the abundance did not change dramatically before (day 0) or after the infection (days 1–7). In the allose-fed group, *Phyllobacterium* consisted of 19.3%–73.1% on average during the course before (day 0) and after post infection (days 0–7), and a slight decrease of the composition ratio (19.3%) was observed on day 1 post-infection with no significant difference compared to the allose-unfed group. The composition ratio of the second most abundant genus, *Leucobacter*, was slightly increased in the allose-unfed group during the course before (day 0) and after post infection (days 0–3), whereas the ratio of the allose-fed group was slightly decreased on day 1 post-infection with no significant difference compared to the allose-unfed group, similar to that of *Phyllobacterium*. Similarly, although the composition ratio of the other four genera—*Staphylococcus*, *Peptococcaceae*, *Proteus*, and *Pseudomonas*—were slightly changed during the course before (day 0) and after infection (days 0–7), the differences between two groups were not significant (**Figure 3B**). In addition, heatmap clustering analysis based on the relative abundance of each genus and beta-diversity analysis showed an allose-independent cluster formation (**Supplementary Figure S1**) and no significant diversity between the allose-fed and allose-unfed groups during the course (**Supplementary Table S1**; PERMANOVA, pseudo-*F* = 1.03, *P* = 0.371), respectively. These results strongly suggested that allose affects the transient growth of midgut microbiota initiated by blood feeding but not their diversity.

### 3.3 Allose did not inhibit growth of bacteria isolated from mosquito midgut

A bacterial growth assay *in vitro* was performed to assess whether allose inhibits the growth of bacteria in the midgut. Two bacteria in the mosquito midgut were successfully isolated and identified as *Leucobacter* sp. and *Phyllobacterium* sp., both of which are dominant in the gut, based on the sequencing analysis of *16S rRNA* fragments with 96.8%–99.7% and 96.4%–99.2% identities, respectively, in the BLASTn analysis. The effect of allose on the growth of these bacteria was assessed *in vitro*. The growth of *Leucobacter* sp. in LB medium containing 100 mM allose and 8% fructose was comparable with that containing 100 mM glucose and 8% fructose as a control (**Table 1**). In addition to the growth rate, the maximum of the growth (stationary phase) in LB medium containing 100 mM allose and 8% fructose was comparable with that containing 8% fructose with 100 mM glucose as a control (**Supplementary Figure S3A**). Similarly, the growth of *Phyllobacterium* sp. in LB medium containing 100 mM allose was comparable with that containing 100 mM glucose as a control (**Table 1**; **Supplementary Figure S3B**) although the growth was inhibited by the presence of 8% fructose in the medium. These results indicated that allose does not have a bactericidal or growth inhibitory effect on these bacteria.

**Table 1:** In vitro growth of Phyllobacterium sp. and Leucobacter sp.

Bacteria	Cultures	Growth rate (OD_600_/h)
*Leucobacter* sp.	LB	0.3459 ± 0.0187
	LB + 8% fructose	0.0507 ± 0.0006
	LB + 8% fructose + 100 mM allose	0.0330 ± 0.0013 a^,b^
	LB + 8% fructose + 100 mM glucose	0.0337 ± 0.0013 a^,b^
*Phyllobacterium* sp.	LB	0.0515 ± 0.0013
	LB + 100 mM allose	0.0352 ± 0.0013 ^c^
	LB + 100 mM glucose	0.0315 ± 0.0017 ^c^

^a^P < 0.01, compared with LB culture in Leucobacter sp.

^b^P < 0.01, compared with LB + 8% fructose culture in Leucobacter sp.

^c^P < 0.01, compared with LB culture in Phyllobacterium sp.

### 3.4 Effect of allose on ookinete development in the mosquito gut

**Figure 4:**
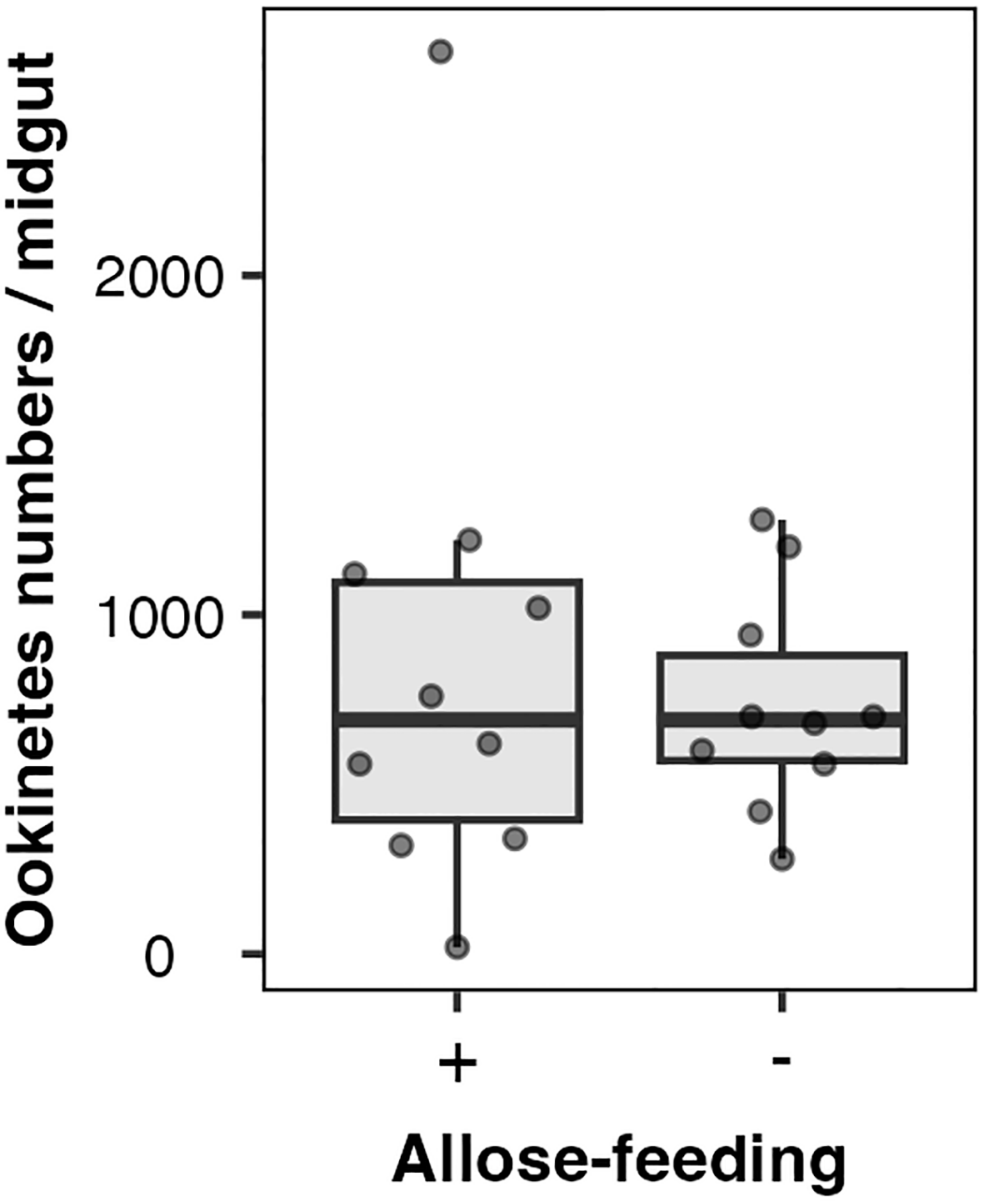
Allose does not inhibit ookinete development in the mosquito midgut. Ten mosquitoes were dissected 24 h after infection, and ookinete count per a midgut was determined. The experiment was repeated three times, and the results are represented as the standard deviation of the mean (SD) of biological replicates.

Allose might impact the development of *Plasmodium* parasites rather than midgut microbiota. The effect of allose on ookinete development within infected mosquitoes was evaluated *ex vivo*. The number of ookinetes in the midgut of allose-fed mosquitoes was comparable to that observed in mosquitoes fed fructose alone at 24 h after infection (**Figure 4**). In addition, ookinetes showing abnormal morphology were not observed in the midgut of allose-fed mosquitoes compared with that in the midgut of allose-unfed mosquitoes (**Supplementary Figure S2**). These results indicated that allose feeding did not inhibit ookinete development of *Plasmodium* parasites at the midgut stage of the infected mosquito.

### 3.5 Exploration of sugars that impact *Plasmodium* development

**Figure 5:**
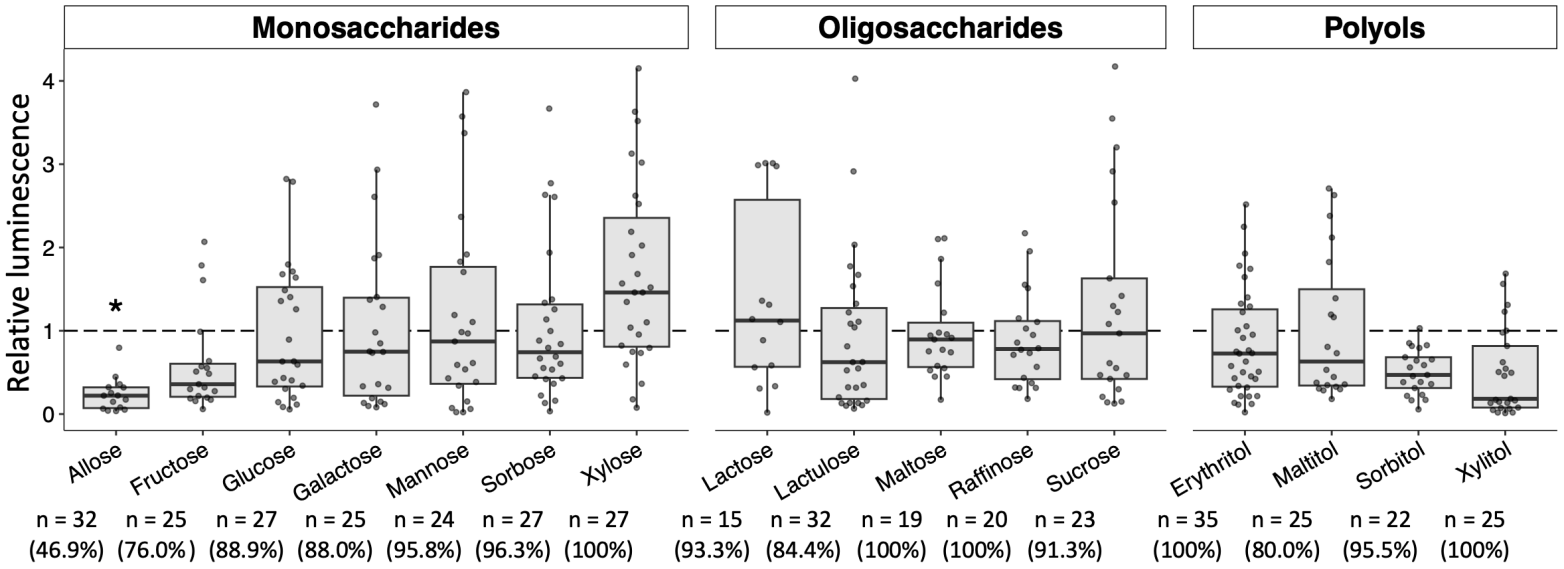
Effect of various sugars on oocyst development in the mosquito midgut. Mosquitoes were fed 8% fructose with 100 mM of each sugar and infected by luciferase-expressing *Plasmodium berghei*. Infectivity was determined by a luciferase assay. Infection of Pb-luc in mosquitoes fed the infected blood after 10 days was determined by measuring luciferase assay. Relative luminescence indicates the luminescence of each explored sugar-fed group normalized by mean luminescence of control group not added their explored sugar in 8% fructose. The number of mosquitoes is indicated under each group. The prevalence of infection was calculated number of detected oocyst or luminescence sample divided by number of total sample. The results represent the standard deviation of the mean (SD) of biological replicates. Statistical significance was determined by Wilcoxon’s rank sum test with Bonferroni’s modification. *P < 0.01.

Additional sugars that impact the development of *Plasmodium* in mosquitoes were explored by targeting the 16 sugars listed above in the Materials and Methods section. Their sugars about the ability to block *Plasmodium* parasites have not been evaluated, except for glucose (Wang et al., 2021). As shown in **Figure 5**, no apparent inhibitory effects on the development of *Plasmodium* in the mosquito were observed with most of the sugars tested. Although fructose, sorbitol, and xylitol slightly inhibited the development of the *Plasmodium* parasite, the inhibition was not significant. These results indicated that these sugars do not appear to have inhibitory effects on the development of *Plasmodium* in mosquitoes.

## 4 Discussion

The midgut stage in the mosquito is one of the bottlenecks in the life cycle of *Plasmodium* parasites, and it can be a target of a transmission-blocking strategy. A previous study showed that allose inhibits oocyst development of *Plasmodium* parasites in the mosquito. However, the underlying mechanism remains unknown (Tokuda et al., 2015). The present study showed that inhibition by allose is effective even when mosquitoes were treated with antibiotics, strongly suggesting that the primary inhibitory mechanism is independent of the midgut microbiota of the mosquito. In addition, ookinete development in the mosquito was not inhibited by allose feeding. These results suggested that allose mainly inhibits development of the parasite after or possibly during its traverse across the midgut epithelium, e.g., ookinete migration across the midgut epithelium, ookinete-to-oocyst developmental transition, and the early phase of oocyst formation.

The interaction of ookinetes with the midgut surface via specific molecules is crucial for the invasion into midgut epithelium (Osta, 2004). Multiple pathways associated with protein–protein interactions between ookinete proteins [enolase, von Willebrand factor A domain–related protein (WARP), membrane-attack ookinete protein (MAOP), *Plasmodium* perforin-like proteins (PPLP5), subtilisin-like protease (SUB2), cell-traversal protein for ookinetes and sporozoites (CelTOS), secreted ookinete adhesive protein (SOAP), and ookinete surface P28 and P25 proteins] and mosquito midgut surface molecules [enolase-binding protein (EBP), aminopeptidase 1 (APN1), annexin-like proteins, carboxypeptidase B, croquemort scavenger receptor homolog, and calreticulin] were suggested to be involved in the invasion process (Siden-Kiamos et al., 2000; Soo Han et al., 2001; Tomas et al., 2001; Yuda et al., 2001; Dessens et al., 2003; Kadota et al., 2004; Kotsyfakis et al., 2005; Kariu et al., 2006; Dinglasan et al., 2007; Ecker et al., 2007; Lavazec et al., 2007; Rodríguez M del et al., 2007; González-Lázaro et al., 2009; Ghosh et al., 2011). In addition, several carbohydrate-binding proteins, i.e., lectins, bind specifically to the midgut epithelial cells of mosquitoes, and carbohydrate moieties are suggested to play a part in the binding of ookinetes to midgut epithelium of the mosquito (Rudin and Hecker, 1989; Zieler et al., 1999; Osta, 2004). Interestingly, *Plasmodium gallinaceum* ookinetes were reported to specifically interact with a carbohydrate ligand on the midgut surface of *Aedes aegypti*, and the specific interaction was competitively blocked by the monosaccharide N-acetylneuraminic acid (Zieler et al., 1999). Therefore, allose may inhibit specific binding of *P. berghei* to midgut epithelium of *Anopheles* mosquitoes. Another possible inhibitory mechanism of allose is related to mosquito immunity against *Plasmodium* parasites. Parasite invasion and development in the midgut is affected by host immunity, including immune-related factors (Blandin et al., 2004; Osta, 2004; Dong et al., 2006; Baxter et al., 2010; Povelones et al., 2011), antimicrobial peptides (Vizioli et al., 2001; Dong et al., 2006), and nitric oxide and ROS (Luckhart et al., 1998; Molina-Cruz et al., 2008). ROS have been shown to have a role in insect innate immune responses as a potent pathogen-killing agent, and allose is reported to induce ROS generation by activating nicotinamide adenine dinucleotide phosphate (NADPH) oxidase through the activity of glucose 6-phosphate dehydrogenase (Kano et al., 2013). RNA interference (RNAi)-mediated silencing of catalase, a ROS detoxification enzyme, was reported to result in decreased *P. berghei* infection (Molina-Cruz et al., 2008) and decreased bacterial load in the mosquito midgut (Bahia et al., 2013). These findings suggest that allose might inhibit the development of *Plasmodium* parasites and affect midgut bacteria via ROS generation in the mosquito midgut.

Several gut symbiotic bacteria, such as *Serratia* spp., *Enterobacter* sp., *Chromobacterium* sp., and *Asaia* sp., are reported to confer resistance to *Plasmodium* infection through activation of mosquito immunity (Cirimotich et al., 2011; Bando et al., 2013; Bahia et al., 2014; Ramirez et al., 2014; Bai et al., 2019; Wang et al., 2021). Initially, we expected that allose may work as a prebiotic for such symbiotic bacteria, resulting in the inhibition of *Plasmodium* development in the mosquito. However, the composition of midgut microbiota in the mosquito was not affected by allose. It seems that allose does not promote the growth of specific bacteria in the gut. In addition, none of the abovementioned bacteria were detected as a major midgut microbiota in our colonized mosquitoes. The microbial community of mosquitoes varies among populations and is associated with locality, climate, and their nutrition (Boissière et al., 2012; Muturi et al., 2018; Sandeu et al., 2022). Because the principal inhibitory mechanism of allose to *Plasmodium* is independent from commensal microbes, allose is expected to inhibit *Plasmodium* development in any population of mosquitoes.

Allose inhibited transient growth of midgut microbiota of mosquitoes initiated by blood feeding without toxic and inhibitory effects on the dominant bacteria. Several probable reasons may explain this observation: 1) allose is not suitable nutrition for midgut microbiota, 2) allose inhibits the bacterial metabolic system necessary for growth, and 3) allose enhances innate immune responses, resulting in the inhibition of bacterial growth. In this study, an equal amount of fructose was given to allose-fed mosquitoes as the control group, and allose was shown to be catabolized in several bacteria such as *Aerobacter aerogenes*, *Escherichia coli*, and *Listeria monocytogenes* (Gibbins and Simpson, 1964; Poulsen et al., 1999; Zhang et al., 2018). In addition, allose did not inhibit the *in vitro* growth of *Leucobacter* sp. and *Phyllobacterium* sp., the dominant bacteria isolated from the mosquito midgut. Recently, modulation of the pH of the mosquito midgut by changing its bacterial composition and metabolites was reported to influence *Plasmodium* gametogenesis (Wang et al., 2021). Our preliminary study showed that allose did not change the pH of the mosquito gut (data not shown). These findings suggested that allose does not directly affect the growth of microbiota in the mosquito gut; rather, the event might be caused by host immunity such as ROS generation.

This study explored 15 additional sugars that might impact the development of *Plasmodium* in mosquitoes. These sugars having various properties were screened, but none of the sugars tested inhibited parasite development, suggesting that allose has unique characteristics. Although pure allose is costly, an affordable and commercially available sweetener, Rare Sugar Sweet (https://www.matsutani.co.jp/english/products/raresugar.html ), which contains D-allose in addition to D-glucose, D-fructose, D-mannose, and another rare sugar, D-psicose, inhibits the development of *Plasmodium* in mosquitoes (Tokuda et al., 2015). Recently, toxic sugar baits were reported to be used as a mosquito control strategy (Fiorenzano et al., 2017); the principle is using fruits and plant as an attractant to attract mosquitoes, but it has some risks for other organisms. Because allose is an eco-friendly natural product, there are fewer limitations to using the attractive toxic sugar bait applications: mixing this sugar instead of an insecticide in a field study to control transmission of *Plasmodium* parasites by mosquitoes.

The present study showed that allose inhibited the development of *Plasmodium* parasites in the mosquito independently of midgut microbiota. Such unique activity appears to be specific to allose. Further study will be needed to disclose the underlying mechanisms, especially focusing on the mosquito immunity elicited by allose. This sugar may be a useful material for vector control of malaria as a “transmission-blocking sugar” in the future (Ferreira et al., 2018).

## Data availability statement

The datasets presented in this study can be found in online repositories. The names of the repository/repositories and accession number(s) can be found in the article/**Supplementary Material**.

## Ethics statement

The animal study was reviewed and approved by Institutional Animal Experiment Committee of Jichi Medical University (approval numbers: 22002-01).

## Author contributions

Conceptualization: DM, MA, and HK. Methodology: DM, DY, AT, MA, and HK. Software: DM. Validation: DM, AT, MA, and HK. Formal analysis: DM. Investigation: DM and MA. Resources: DM, DY, AT, MA, and HK. Writing—original draft preparation, DM and HK. Writing—review and editing: DM, DSY, AT, MA, and HK; visualization: DM; supervision: AM and HK; project administration: DM and HK. Funding acquisition: DM and HK. All authors have read and agreed to the published version of the manuscript.
