# Supplementary material for: A rare sugar, allose, inhibits the development of Plasmodium parasites in the Anopheles mosquito independently of midgut microbiota
Source: Front Cell Infect Microbiol. 2023 Jul 20;13:1162918. doi: 10.3389/fcimb.2023.1162918 (PMC10400720; doi:10.3389/fcimb.2023.1162918)
Supplement: Supplementary file 1 [file DataSheet_1.docx]

Supplementary Material

A rare sugar, allose, inhibits the development of *Plasmodium* parasites in the *Anopheles* mosquito independently of midgut microbiota

Daiki Mizushima^*^, Daisuke S. Yamamoto, Ahmed Tabbabi, Meiji Arai and Hirotomo Kato

*** Correspondence:** Daiki Mizushima: dmizushima@jichi.ac.jp

# Supplementary Figures and Tables

## Supplementary Figure S1

**Supplementary Figure S1.** Heatmap clustering analysis of genera based on relative abundance in midgut microbiota from allose-fed and unfed mosquitoes. The heatmap clustering analysis was performed with the relative abundance of each genus in the samples. Euclidean distance was applied to the hierarchical clustering using the gplots package with R software version 4. 0. 3.

## Supplementary Figure S2

**Supplementary Figure S2.** Morphology of ookinetes in the midgut of mosquitoes fed on fructose with or without allose. The upper and lower panels show the ookinetes in the midgut of mosquitoes fed on fructose alone (–) and fructose with allose (+), respectively. Scale bar = 5 µm.

## Supplementary Figures S3

**Supplementary Figure S3.** Effect of allose on the growth of *Leucobacter* sp. and *Phyllobacterium* sp.. *Leucobacter* sp. **(A)** and *Phyllobacterium* sp. **(B)** isolated from mosquito gut were cultured *in vitro* in the presence or absence of allose, and the inhibitory effect of allose on bacterial growth was determined. **(A)** LB medium alone (gray line) and LB plus fructose (green line), fructose and allose (red line), or fructose and glucose (blue line) were used as the medium for *Leucobacter* sp. **(B)** LB medium alone (gray line) and LB plus allose (red line) or glucose (blue line) were used as the medium for *Phyllobacterium* sp. Error bars indicate standard deviation (n = 3).

## Supplementary Figure S4

**Supplementary Figure S4.** Allose inhibits oocyst development in the mosquito midgut. The represented figure is the replicated result of figure 1. Allose-fed and -unfed mosquitoes were infected by luciferase-expressing *Plasmodium* *berghei*, and infectivity was determined 10 days post-infection by counting of the number of oocysts and by measuring the luciferase activity. The number of mosquitoes is indicated under each group. The prevalence of infection was calculated number of detected oocyst or luminescence sample divided by number of total sample. Statistical significance was determined by Wilcoxon’s rank sum test with Bonferroni’s modification. * *P* < 0.01.

## Supplementary Table S1

**Supplementary Table S1.** Qualification of the 16S ribosome RNA gene amplicon sequence in allose-fed mosquito midgut. “Stats” sheet shows the number of qualified sequences for microbiome composition analysis in each sample. “Summarized table” sheet shows the feature ID, taxon, frequency (number of reads), sequence, and sequence length in each ASV. “Unassigned_blastn” sheet shows the results of BLASTn search for the unassigned ASVs sequences. Organism names of the subject sequence highest match with the unassigned query sequence are described in the description column. “Ricke._mit._blastn” sheet shows the results of BLASTn search for the *Mitochondria* ASVs sequences. Organism names of the subject sequence highest match with the unassigned query sequence are described in the description column. “*Microbacteriaceae*_blastn” sheet shows the results of BLASTn search for the *Microbacteriaceae* ASVs sequences. Organism names of the subject sequence highest match with the unassigned query sequence are described in the description column. “Taxa_sample_matrix” sheet shows a comparison matrix of frequencies between taxonomies and samples.
